# Course of depression symptoms between 3 and 8 months after delivery using two screening tools (EPDS and HSCL-10) on a sample of Sudanese women in Khartoum state

**DOI:** 10.1186/s12884-018-1948-1

**Published:** 2018-08-08

**Authors:** Dina Sami Khalifa, Kari Glavin, Espen Bjertness, Lars Lien

**Affiliations:** 1 Department of Community Medicine, Institute of Health and Society, Faculty of Medicine, University of Oslo, Postboks 1130 Blindern, 0318 Oslo, Norway; 2 Faculty of Health Sciences, Ahfad University for Women, Khartoum, Sudan; 3 VID Specialized University, Oslo, Norway; 4 National Advisory Board on Dual Diagnosis, Innlandet Hospital Trust, Hamar, Norway; 5 Department of Public Health, Hedmark University College, Elverum, Norway

**Keywords:** Postnatal depression, Course of depression, Maternal distress, Screening, EPDS, HSCL-10

## Abstract

**Background:**

Effects of depression on parenting and on cognitive development of newborns are augmented when symptoms continue throughout the first postnatal year. Current classification systems recognize maternal depression as postnatal if symptoms commence within four to six weeks. Traditional cultural rituals in Sudan offer new mothers adequate family support in the first 6–8 weeks postpartum. The course of postnatal depression symptoms beyond that period is not explored in such settings. We therefore aim to investigate the change in screening status and in severity of depression and distress symptoms between three and eight months postpartum among a sample of Sudanese women using the Edinburgh Postnatal Depression Scale (EPDS) and a locally used tool: the 10-items Hopkins Symptoms Checklist (HSCL-10).

**Methods:**

Three hundred pregnant women in their 2nd or 3rd trimester were recruited from two clinics in Khartoum state. They were followed up and screened for depression symptoms eight months after delivery by EPDS at ≥12, and by HSCL-10 at ≥1.85. The same sample was previously screened for depression at three months after birth.

**Results:**

Prevalence of postnatal depression symptoms by EPDS was lower at eight months compared to three months after birth (3.6% at eight months (8/223) compared to 9.2% at three months (22/238), *p* <  0.001). Eight Mothers exhibited depression symptoms eight months after birth. Depressed mothers at three months had a 56% reduction in EPDS mean scores by eight months and 96.4% of participants either remained in the same EPDS category, or improved eight months after birth. Four participants with major depression symptoms at eight months were also depressed three months after birth and four participants had new onset depression symptoms. The HSCL-10 measured higher distress than EPDS across the two screening points (19.3% at three months, 9.1% at eight months postpartum, *p* <  0.001). Nonetheless, the two tests correlated positively at both points.

**Conclusions:**

Repeated screenings by EPDS (depression surveillance) is recommended during the first postnatal year because a subset of mothers can have symptoms beyond the early postnatal period. Existing depression screening instruments can be assessed for their validity to detect PND.

## Background

Depression is classified as postnatal depression (PND) by the 10th revision of the International Statistical Classification of Diseases and Related Health Problems (ICD-10) if symptoms occur within the first six weeks after birth, and by the Diagnostic and Statistical manual for Mental Disorders (DSM) if symptoms occur within the first four weeks after birth [1, 2]. Postnatal depression affects a mother’s capacity to form and maintain attachment with her newborn increasing the risk of attachment disorders during infancy and childhood [3]. Compared to a previous report by the World Health Organization (WHO) on PND in low and lower middle income countries (LLMIC) [4], recent reviews on prevalence of postnatal depression show higher estimates. Norhayati et al. [5] reported from LLMIC, based on screening results by the Edinburgh Postnatal Depression Scale (EPDS), prevalence estimates ranging from 12.9 to 50.7% at less than four weeks screening, 4.9–50.8% at four to eight weeks, 8.2–38.2% at six months after birth and 21.0–33.2% in the first postnatal year.

Prolonged postnatal depression is linked to maternal morbidity and to poor childhood cognitive and behavioral development [6]. Minkovitz et al. [7] followed children in the US up to age three and reported that mothers diagnosed with postnatal depression accessed well-baby and immunization visits at a lesser rate than mothers without depression, and they utilized emergency services for their infants at a higher rate. A 16 months US follow-up study on infant and maternal health-related quality of life by Darcy et al. [8] reported that maternal depressive symptoms at four months after birth predicted poorer infant health at 8, 12 and 16 months assessments. O’Brian et al. [9] examined two years old children in the UK and reported that those with faltering growth are more likely to have depressed mothers than children who are gaining weight appropriately. These findings elucidate the risk of continuing or “chronic depression” on health of children due to longer periods of maternal negative affects. The well-documented evidence on impact of PND on the quality of life of both mother and child makes it logical to identify and treat it. Wickramaratne et al. [10] reported that early remission of maternal depression leads to improvement in all child outcomes.

Many health systems worldwide advocate for universal screening for postnatal depression to increase detection rate, but the optimum time for screening or the frequency of screening needed during the postnatal period is still unclear. This issue has been raised by few scholars [11–13]. The DSM 4 and 5, as well as the ICD-10, do not classify late onset postnatal depression [2, 14]. For that reason, exploration of the course of depressive symptoms after birth has become paramount.

A number of systematic reviews and longitudinal studies illustrated the course of depression symptoms after birth. Halbreich and Karkun [15] reviewed studies from high and low income countries. They showed that higher estimates of depression prevalence are commonly noticed during periods closer to birth than periods further away when brief instruments are used for screening, mostly EPDS [15]. Goodman’s [16] study reviewed PND beyond the early postnatal period (from 6 months up to 2 1/2 years after delivery) in high-income countries and reported that a significant proportion of mothers continue with depressive symptoms for months and years after giving birth. Vliegen et al. [17] reported on seven longitudinal studies that followed mothers up to three years after birth in high-income countries. The review showed a general reduction in severity of depression symptoms throughout pregnancy and the postnatal period, but not all reductions were statistically significant [17]. They also reported prevalence estimates from 20 longitudinal studies on community-based and clinical samples. They concluded that 30% of depressed mothers from community samples and 50% of depressed mothers from clinical samples remain depressed throughout and beyond the first postnatal year [17].

Studies from the US also reported continued symptoms of depression one and two years after birth [18, 19]. A study from Pakistan recruited 701 mothers during their third trimester of pregnancy and assessed them for depression at 3, 6 and 12 months after birth [20]. Fifty-six percent of mothers that completed follow-up exhibited depression symptoms at all points of assessment [20].

We have previously reported on the three months prevalence of PND symptoms using the EPDS [21]. We have little knowledge on how postnatal depressive symptoms evolve in a North African context: how many cases have a more prolonged course and will new cases appear after the three months period? In addition, will those new cases be “normal” depressive episodes or late postnatal depression? There is also a need to find out if other screening instruments for depressive symptoms used locally are useful in the detection of symptoms in the postnatal period.

The objective of this article is to investigate the course of depressive symptoms up to eight months after birth in a Sudanese sample using the Edinburgh Postnatal Depression Scale (EPDS). In addition, we want to explore correlations between the EPDS and a locally used tool for screening for depression outside pregnancy: the 10-items Hopkins Symptoms Checklist (HSCL-10) points.

## Methods

### Study design

This is a follow-up study of 300 women recruited during pregnancy. Women attending two antenatal clinics (ANC) in two major public tertiary hospitals consented to participate in the study. They were screened for symptoms of postnatal depression at three and eight months by EPDS and HSCL-10. The clinics provide routine antenatal care services for pregnant women living within or outside the hospitals’ catchment population. The hospitals were Omdurman Maternity Teaching Hospital and Ibrahim Malik Teaching Hospital. Compared to other states in Sudan, Khartoum state has the highest level of utilization of ANC services and the highest level of institutional deliveries as well [22]. Antenatal care attendance in Khartoum state is 88% [22]. This is the proportion of women that attend “at least one” ANC visit provided by a skilled provider. Women from all localities of Khartoum state can access services in Omdurman Maternity Hospital irrespective of their residence [23]. The sample size was calculated using the prevalence of PND in Nigeria, an African country with a similar social context to Sudan [24]. Inclusion criteria were women of Sudanese nationality, in their 2nd or 3rd trimester, of any parity and with full contact information (at least two working telephone numbers). Availability of two working phone numbers was imperative to improve follow-up rates through house visits, as the address system in that context was unclear. Illiteracy was not an exclusion criterion as data collection was via interviews. The study protocol was approved in Sudan by the Sudan Ministry of Health and in Norway by REK (Regional Committees for Medical and Health Research Ethics, reference no. 2013/353/REK).

### Study procedure

**Fig. 1:**
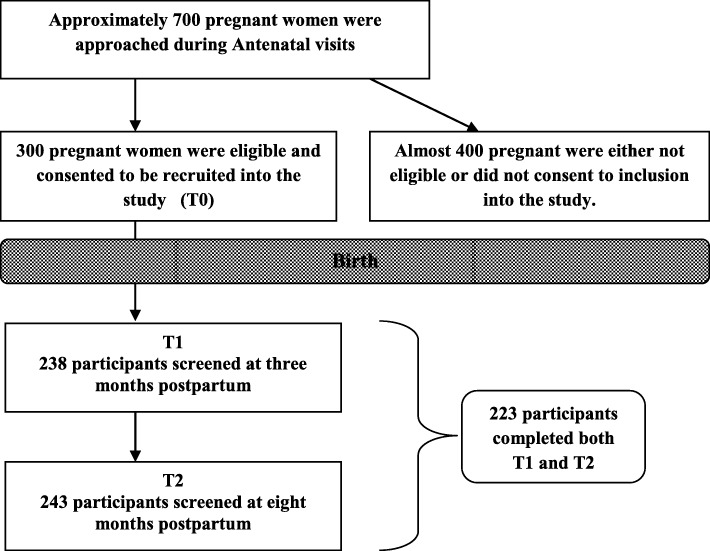
A flow chart illustrating the follow-up process

Recruitment was intermittent during the period of April 2013 until April 2014. More than 5000 women attended the clinics during that period; the principle investigator approached approximately 700 women. The attending physician screened attendants for the required gestational age and introduced the investigator to each prospective participant at the end of her ANC visit. Random sampling from a list of ANC attendees was not possible in that setting. Approximately four hundred women were not included: almost two hundred women refused to be part of a research study and the remaining were excluded due to none eligibility because they had no telephone number. No information was available of those who refused participation. Recruitment continued during that period until 300 women consented to participate in the study. They were interviewed at recruitment (T0), and screened at three months (T1) and eight months postpartum (T2). Figure 1 illustrates the follow-up process.

#### First interview (T0, *n* = 300)

Full contact information was obtained at recruitment to optimize follow-up and screening rate for PND after delivery. Median age of women at recruitment was 28 years (range 15 to 43); 41% were between the ages of 15–25. Twelve percent held an occupation. The majority (215 women) had no previous employment (72%), 10% (31 women) had an occupation before marriage or childbirth, and 6% (17 women) were students. [21]

#### Second interview (first screening) (T1, *n* = 238)

At three months postpartum, participants were interviewed regarding circumstances of the index pregnancy. The interview was either face-to-face (at home or ANC clinic), or through phone. Phone interviews were conducted to minimize loss of follow-up only when women were away from Khartoum state or refused home interviews. The first EPDS and HSCL-10 screening was conducted at that time. A single interviewer conducted the interviews. As reported from a previous analysis in the same study [21], the response rate at T1 was 79.3% (62 participants were lost to follow-up). The loss to follow-up was due to personal refusal (14 participants), husband’s refusal (13 participants), and contact failure (35 participants). Participants lost to follow-up were not significantly different from participants who completed the follow-up in age (the median age was 27 years old for both groups), in parity (the median parity was 1.9 children and 1.8 children, respectively) or in educational level (Pearson chi-square *p*-value = 0.70) [21].

#### Third interview (second screening) (T2, *n* = 243)

At eight months postpartum, 243 women were screened for PND with EPDS and HSCL-10 resulting in a follow-up rate of 81%. Fifty-seven women were lost in the second screening due to contact failure (27 participants), personal refusal (15 participants), and husband refusal (15 participants).

### Measurement tools

#### EPDS

The Edinburgh Postnatal Depression Scale (EPDS) is a self-reporting tool specifically developed for screening for symptoms of postnatal depression at primary healthcare level [25]. It has been translated and validated into 57 languages including Arabic [26–28]. We have validated the Arabic EPDS in this sample and we have described its validity indices against a diagnostic tool [21]. The EPDS screens for PND through ten inventory questions investigating new feelings felt by the mother within the previous seven days. Each question has four possible answers rated from 0 to 3 and the scale has a total score of 30. In this study, EPDS was administered through personal interviews and a test is “positive” for major depression if the woman scores 12 or more out of 30 as set by Cox et al. [25]. A cut-off point of ≥10 is optimum for screening for minor and major depression combined [25]. Combined subscale analysis of EPDS confirms that there is an anxiety scale embedded within the tool and that the whole 10 item tool measures both depression and anxiety [29].

Although it is a self-administered tool, studies have shown that administering EPDS through directed interviews is an equivalent screening technique [30]. According to Ghubash et al. [27], the Arabic EPDS has good internal consistency and reliability with a Cronbach’s coefficient of 0.84. In the current study, the Cronbach’s coefficient was 0.83.

Prevalence of PND symptoms at T1 with the EPDS at ≥12 was 9.2% [21]. Validity indices of the EPDS were 89% sensitivity, 82% specificity, 98.7% NPV and 33% PPV [21].

Only a subsample of participants was clinically interviewed for assessment of their depression symptoms (the EPDS “test positives” at T1 and their matched controls). Clinically depressed participants were referred to the outpatient mental health clinic in Khartoum for further management.

#### HSCL-10

Originally the Hopkins Symptoms Checklist was a 58-item self-reporting inventory symptom checklist developed in the mid-1970s for psychological distress [31]. A ten items version as well as 35, 25 and five items versions were also developed and validated against the extended version and were found of equal performance [32, 33]. The first four items in the 10-item tool evaluate “anxiety” and the remaining six items “depression”. Each item has a 4-point scale ranging from “not at all” to “extremely”. The symptoms screened were in the seven days prior to screening. HSCL-10 final score is the average of the total score. The cut-off score for “distress” used in this study is ≥1.85 [32]. It has been translated to Arabic and validated on Arab speaking populations [34]. HSCL-10 is brief and simple. It has well-documented reliability and validity and is an easily administered instrument. In this analysis, it has an acceptable internal consistency of 0.77.

### Statistical analysis

Prevalence of major depression symptoms at eight months postpartum (T2) was calculated based on EPDS at a cut-off point ≥12/30. The prevalence of depression symptoms at T1 and T2 with EPDS at a cut-off score ≥ 10/30 was also explored (reflecting prevalence of major and minor depression combined).

Prevalence of psychological distress with HSCL-10 at three months (T1) and eight months (T2) postpartum was calculated at a cut-off point ≥1.85. Correlation coefficients among scores of the two tests were computed. Numbers of new and continuous depression symptoms between the two screening points, based on EPDS, were calculated. The change in EPDS test status between T1 to T2 was analysed for its statistical significance (i.e. if individual scores and mean scores fell below cut-off score) and for its clinical reliability using the Reliability Change Index method (RCI) as described by Jacobson and Truax [35]. This analysis is on data of participants that completed follow-up at T1 *and* T2 (i.e. participants with complete follow-up).

## Results

Participants lost to follow-up at the eight months’ screening were not significantly different from women that were screened in terms of median age; 27 years old and 28 years old respectively, in parity (2 children and 1.8 children, respectively, *p*-value = 0.48) or in educational level (Pearson chi square *p*-value = 0.78).

Participants with complete follow-up at T1 and T2 were 223 (74.3% of sample) and 77 participants did not complete screening either at T1 or at T2 (see Fig. 1). Mean scores of EPDS at T1 and T2 for the entire sample were compared with mean scores for the 223 participants. Differences in mean scores (between 0 and 0.06 points on a 30-point scale) were insignificant between participants that had complete follow-up and participants that were lost at either T1 or T2. Table 1 illustrates the characteristics of women that completed follow-up.

**Table 1:** Characteristics of women with complete follow-up (*n* = 223)

Variable	No. (%)
Educational level	
University and Postgraduate	89 (39.9%)
Secondary	64 (28.7%)
Primary	65 (29.1%)
No education	5 (2.2%)
Parity	
Primigravida	56 (25.1%)
Multigravida(1–4)	147 (65.9%)
Grandmultipara (= > 5)	20 (9%)
Polygamy	
Yes	17 (7.6%)
No	206 (92.4%)
Place of delivery	
Health Facility	206 (92.4%)
Home	17 (7.6%)
Mode of delivery	
Vaginal (incl. abortion)	139 (62.3%)
C/section	84 (37.7%)
History of psychological condition	
Yes	18 (8.1%)
No	205 (91.9%)
Family history of a psychological condition	
Yes	37(16.6%)
No	186(83.4%)

### Course of postnatal depression symptoms among women with “complete follow up”

**Fig. 2:**
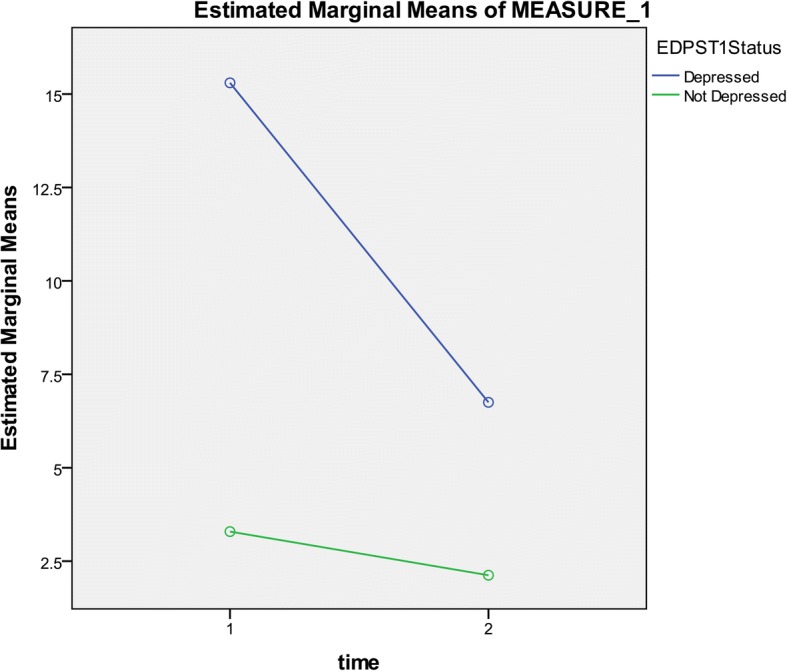
Change of mean EPDS scores from T1 until T2

Twenty participants at three months and eight participants at eight months tested “positive” by EPDS at a cut-off score of ≥12 (correlation coefficient = 0.57; *p* < 0.001). To reflect minor and major depression combined, 26 participants at three months and 10 participants at eight months scored ≥10 cut-off score. A paired-samples t-test was conducted on EPDS scores across T1 and T2. There was a significant reduction of EPDS mean scores between T1 and T2 (Table 2). A mixed between-within subjects ANOVA was conducted to assess the effect of time on depression status. Figure 2 illustrates the change in mean scores over time for EPDS test “positives” and “negatives” from T1 until T2. There was a significant interaction between time and depression status (Wilks’ Lambda = 0.69, F (1,221) =98.9, *p* < .0005, partial eta squared = 0.31) meaning that the change of scores across time was different between depressed and non-depressed mothers. Mothers with depression symptoms at three months were more likely to recover by eight months. Their mean EPDS score was 15.3 (SD = 3.2) at T1 and 6.7 (SD = 5.7) at T2. There was 56% reduction in mean scores to below the cut-off score of <12. Non-depressed mothers at three months showed stable symptomology up to eight months after birth.

**Table 2:** Prevalence of depression and distress symptoms at 3 months (T1) and 8 months (T2) postpartum with EPDS and HSCL-10 (*n* = 223)

	Prevalence	Mean of scores(SD)	Difference of means	SD	95% CI	*P* value
EPDS T1^a^	9.2%	4.37(4.5)	1.83	3.8	1.33–2.33	< 0.001
EPDS T2	3.6%	2.54(3.3)				
HSCL-10T1^b^	13.5%	1.44(0.38)	0.13	0.32	0.08–0.16	< 0.001
HSCL-10 T2	5.8%	1.31(0.28)				

^a^Cut-off score ≥ 12/30

^b^Cut-off score ≥ 1.85/4

**Fig. 3:**
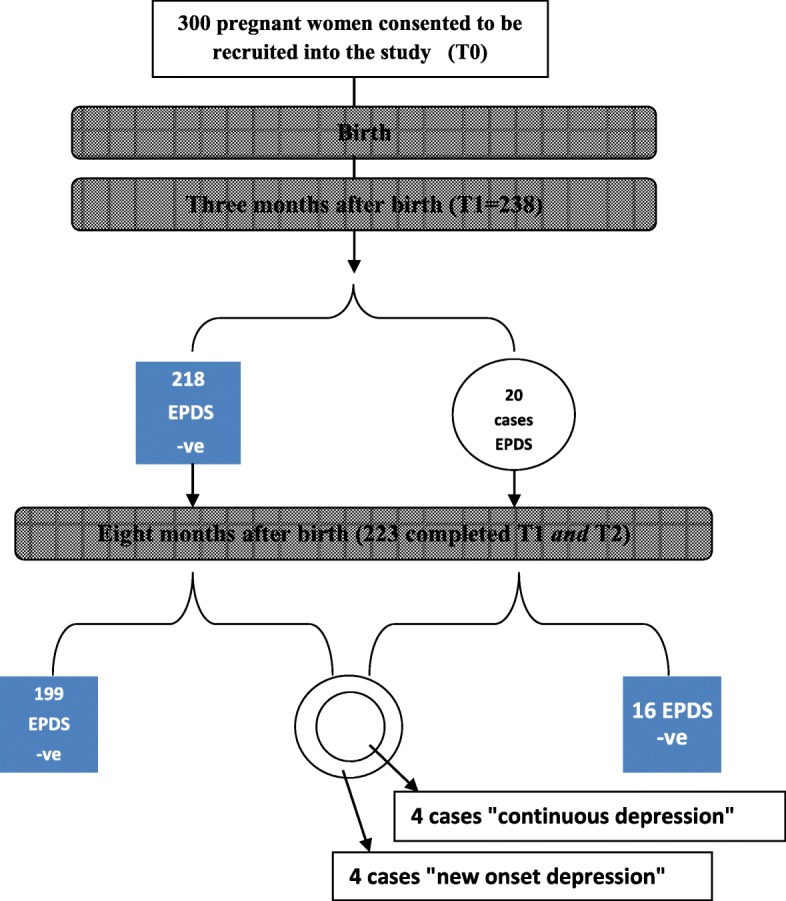
Results of the EPDS screening tests at the two screening points for participants with complete follow-up

The next set of analysis was about EPDS “test positives” at T1 (20 women) (see Fig. 3). Sixteen out of those 20 women scored below 12 at T2. The Reliable Change Index Score for each of the 16 was more than 1.96 (two standard deviations) indicating reliable change. They constitute the remission group. The remaining four participants experienced “continuing depression” The Reliable Change Index Score for each was below 1.96 indicating unreliable change (i.e. we are 95% confident that change in EPDS scores was not real and most likely due to measurement error). All four “continuing depression” participants were clinically diagnosed with depression at T1 by a diagnostic interview and all had corresponding scores above ≥1.85 by HSCL-10 at both screening points. Their ages were 23, 26, 27 and 30 years, Two had a history of domestic violence, one had a perceived history of depression before pregnancy. One participant suffered from anemia during pregnancy and all had term pregnancies. With regard to newborn illness: one participant had a newborn suffering from a congenital anomaly. Three participants had primary level education and one was a university student.

In addition, four other participants developed *new* major depressive symptoms at eight months after delivery (EPDS score was ≥12 at T2). They constitute the “incident” depression group. Their ages were 28 (2 women), 32 and 37 years. None of them had a history of domestic violence or of a perceived past psychological condition. One suffered a stillbirth. Otherwise, their medical and obstetrical history was unremarkable. Mothers with new onset symptoms at 8 months seem to have higher parity compared to mothers with continuing depression symptoms (the former had two, four, and six children compared to no children, one and two children). Figure 3 illustrates the results of the EPDS across the two screening points.

The majority of the respondents (89.2%) remained in the same EPDS category across the two time points (always scoring <12 in EPDS). In fact, 96.4% of participants either remained in the same EPDS category, or improved eight months after birth.

Only three participants diagnosed with clinical depression by a clinical interview at T1 responded to our referral to the local psychiatric outpatient clinic and received appropriate management. One of them was lost to follow-up at 8 months and the other two participants scored below 12 at 8 months.

### Prevalence of maternal distress by HSCL-10 at the two screening points

At a cut-off point of ≥1.85, HSCL-10 measured higher distress among participants than EPDS. At three months after birth, 30 participants (13.5%) had high distress symptoms. At eight months screening (T2) 13participants (5.8%) had high distress (Table 2). A paired-samples t-test was performed on HSCL-10 scores on T1 and T2. Mean scores of HSCL-10 significantly decreased by 8.3% at T2 for all 223 participants (Table 2).

There were strong and positive correlations between depression and distress at the two screening points (Table 3). Correlations between EPDS total scores and the two anxiety and depression subscales of HSCL-10 were similarly positive and significant but stronger at T1 than T2. Higher scores of HSCL-10 are strongly associated with higher scores of EPDS, and HSCL-10 explained almost 60% of the depression symptoms detected by the EPDS in the sample.

**Table 3:** Correlations between EPDS & HSCL-10 at 3 and 8 months postpartum

	Correlation with EPDS	*P* value^a^
At T1 (3 months postpartum)		
Total HSCL-10 score	0.77	<.001
Depression subscale	0.69	
Anxiety subscale	0.67	
At T2 (8 months postpartum)		
Total HSCL-10	0.78	<.001
Depression subscale	0.59	
Anxiety subscale	0.59	

^a^*P* value is for Pearson correlation coefficient

## Discussion

The study illustrates that depression symptoms can exist well beyond the early postnatal period on a subset of women. The study also showed a general reduction of severity of symptoms for most mothers further away from birth. The decrease in the level of depression symptoms was probably not due to active treatment of clinically diagnosed cases, as very few proactively sought our referral services. We also showed that depression at three months constitutes higher risk and could predict depression later on in the first postnatal year. These findings were paralleled by both screening tests: all mothers reporting high depressive symptoms also showed a similar pattern of heightened distress Heron et al. [36] assessed the pattern of symptoms in a large community sample in England (*n* = 8323) during pregnancy up to 8 months after birth by self-reporting tools [37]. They reported a mean decrease in depression and anxiety across the period. McMahon et al. followed 100 postnatal mothers in Australia and measured depression symptoms by self-reporting tools one year after delivery [37]. They reported that 30% of all mothers and 60% of those depressed at 4 months continued to report significant depression at one year [37]. They also reported on factors predicting the persistence of depressive symptoms. Horowitz et al. [38] followed mothers that had high depression symptoms when screened 2 to 4 weeks after birth. Two years after birth, 30% of these mothers continued with depressive symptoms [38]. Women with a chronic course of depression were more likely to have suffered from previous depression, have high parental distress and limited current partner support [38]. Depression symptoms with atypical time of onset or course have raised the argument whether symptoms are in fact PND or an undiagnosed bipolar disorder that was triggered by the birth experience. Recent studies have explored the possibility of PND being an indicator for underlying bipolar depression. Liu et al. [39] conducted a Danish register-based cohort study and reported that the risk of bipolar depression among women with postnatal depression symptoms was higher than in women with depression outside the first year after birth. Sharma et al. [40] reported that 21.4–54% of women with PND in different studies have a diagnosis of bipolar depression. Having a mood disorder during such sensitive periods has tremendous impact on the mother, child, partner, and family. A chronic course of depression will involve more impairment and role limitations for depressed mothers. The period during and after pregnancy in low resource settings is a time of highest contact between mothers and healthcare services and it is a missed opportunity to detect and manage depression symptoms, whether it is unipolar or bipolar.

This article adds to existing research from North Africa by taking a prospective approach, measuring, and monitoring the course of depression and distress symptoms after the customary period for postnatal screening. This analysis highlights to healthcare personnel that they should not rule out PND if symptoms occur after the early postnatal period [41]. Women may not recognize the symptoms and may not seek help in the immediate weeks following birth. There are still limits in existing classifications of postnatal depression by the Diagnostic and Statistical Manual for Mental disorders (DSM 4 and 5) and in ICD-10 in spite of clear evidence from studies on late postnatal onset of depression [41–43]. Nonetheless, a depression screening strategy should take into account potential relapse or chronic course of depression symptoms. Women with relevant risk factors are more likely to have a consistent level of depression symptoms throughout the first postnatal year [44]. Hence, it will be cost-effective, in low resource settings, to redirect repeated screening and follow-up care to women at higher risk of depression, as the number of women may be small. Low risk mothers will be reached through health promotion and awareness raising, even if they are not targeted for repeated screening for depression. That way they are equipped with knowledge if they do suffer from symptoms during pregnancy or after birth. To prevent a chronic course of postnatal depression, it may be necessary to identify symptoms and risk factors as well [44]. As our study showed, a negative early screening test may predict a continued negative test later on and this reduces the need for repeated screening for all women.

The study suggests that existing screening tools used to screen for depression can correlate with tools developed to detect women at risk for PND, especially in low resource settings where the possibility of adopting new tools is difficult. Further studies to compare performance of HSCL-10 against a diagnostic tool are needed to optimize cut-off points suitable for the perinatal period. We also recommend further qualitative studies on why women are reluctant to respond to referrals to mental health services.

Our study had several limitations. We relied on self-reporting instruments, not on clinical interviews, to measure severity and course of symptoms. In addition, the study did not investigate onset of depression symptoms during pregnancy. We missed the opportunity to assess if antenatal depression predicted the prevalence and course of postnatal depression. Due to this limitation, we illustrated our results as new onset late postnatal depression. Also, repeated screening by the investigator may have provided participants with some supportive talk therapy that could have biased (underestimated) our eight months screening results. Studies have shown that the most desired method of treatment by women feeling depressed is talking about their feelings with a sympathetic listener who understands the nature and extent of their feelings [45]. On the other hand, the concept of repeated screening may have raised feelings of stigma or intrusion among our respondents. This may have increased the number of participants who ignored or refused our second screening, although this risk was investigated statistically and was proved minimal. In addition, we do not consider our estimates generalizable to all women in Sudan. Our estimates may underestimate the magnitude of PND among mothers, for example, in the Eastern and Southern regions of Sudan, which are states suffering from political unrest and other psychosocial risk factors.

## Conclusion

The ICD-10 and DSM-5 PND onset classification does not reflect consistent evidence brought about from longitudinal studies. Hence, postnatal depression will remain underdiagnosed with consequent policy implications. Screening strategies should advocate repeated screening during the first postnatal year as symptoms could persist or relapse beyond the early postnatal period. In low resource settings, repeated screening could be directed to mothers with relevant risk factors for depression.

## Abbreviations

ANC: Antenatal care
CI: Confidence interval
DSM: The Diagnostic and Statistical Manual for Mental disorders
EPDS: Edinburg Postnatal Depression Scale
HSCL: Hopkins Symptoms Checklist
ICD: International Statistical Classification of Diseases and Related Health Problems
LLMIC: Low and lower middle income countries
NPV: Negative protective value
PND: Postnatal depression
PPV: Positive predictive value
REK: Regional Committees for Medical and Health Research Ethics
WHO: World Health Organization
